# A novel microtubule nucleation pathway for meiotic spindle assembly in oocytes

**DOI:** 10.1083/jcb.201803172

**Published:** 2018-10-01

**Authors:** Pierre Romé, Hiroyuki Ohkura

**Affiliations:** Wellcome Centre for Cell Biology, School of Biological Sciences, University of Edinburgh, Scotland, UK

## Abstract

The meiotic spindle in oocytes is assembled in the absence of centrosomes, the major microtubule nucleation sites in mitotic and male meiotic cells. A crucial, yet unresolved question in meiosis is how spindle microtubules are generated without centrosomes and only around chromosomes in the exceptionally large volume of oocytes. Here we report a novel oocyte-specific microtubule nucleation pathway that is essential for assembling most spindle microtubules complementarily with the Augmin pathway. This pathway is mediated by the kinesin-6 Subito/MKlp2, which recruits the γ-tubulin complex to the spindle equator to nucleate microtubules in *Drosophila* oocytes. Away from chromosomes, Subito interaction with the γ-tubulin complex is suppressed by its N-terminal region to prevent ectopic microtubule assembly in oocytes. We further demonstrate in vitro that the Subito complex from ovaries can nucleate microtubules from pure tubulin dimers. Collectively, microtubule nucleation regulated by Subito drives spatially restricted spindle assembly in oocytes.

## Abstract (precis)

Romé and Ohkura show that the kinesin-6 Subito/MKlp2 mediates a novel oocyte-specific microtubule nucleation pathway, which is essential for assembling spindle microtubules complementarily with the Augmin pathway.

## Introduction

Spatial and temporal regulation of microtubule nucleation is vital for the formation and maintenance of a functional spindle. In mitotic or male meiotic animal cells, centrosomes are the major microtubule nucleation sites, which appear to be central in defining the position of the spindle formation and the spindle bipolarity. Despite the apparent central roles of centrosomes, the bipolar mitotic spindle can be formed in mitotic cells even when centrosomes are artificially inactivated (Khodjakov et al., 2000; Hinchcliffe et al., 2001). Moreover, centrosomes have been shown to be dispensable for the flies, as *Drosophila melanogaster* without centrosomes can survive to the adult stage (Basto et al., 2006).

In most animals, female meiosis in oocytes is different from mitosis or male meiosis, as oocytes naturally lack centrosomes to assemble the meiotic spindle (McKim and Hawley, 1995; Karsenti and Vernos, 2001). The lack of centrosomes in oocytes poses two fundamental questions: How are spindle microtubules nucleated, and how is efficient nucleation spatially restricted to only around the chromosomes? A constantly high level of nucleation is essential for forming and maintaining the spindle, due to the intrinsically dynamic nature of spindle microtubules (Mitchison and Kirschner, 1984). In addition, as oocytes are exceptionally large in volume, it is crucial to spatially limit a high level of nucleation only to the vicinity of chromosomes.

In mitotic cells, centrosome-independent microtubule nucleation takes place randomly along the spindle microtubules (Mahoney et al., 2006). This nucleation has been shown to be mediated by a conserved 8-subunit complex called Augmin (Goshima et al., 2008; Meireles et al., 2009; Uehara et al., 2009; Kamasaki et al., 2013). Augmin sits on a preexisting microtubule and recruits the γ-tubulin complex by direct interaction with a component of the γ-tubulin ring complex, NEDD1 (Grip71 in *Drosophila*) (Uehara et al., 2009; Chen et al., 2017). The γ-tubulin complex then nucleates a new microtubule from a preexisting one, leading to “microtubule amplification” which exponentially increases the number of microtubules (Goshima and Kimura, 2010). In mitotic cells, a loss of either Augmin or Grip71 leads to the same dramatic decrease in the spindle microtubule density (Meireles et al., 2009; Reschen et al., 2012), demonstrating that this microtubule amplification pathway is responsible for the majority of microtubule assembly even in the presence of centrosomes. Furthermore, loss of Augmin leads to severe reduction of spindle microtubules in mitotic cells which lack centrosomes (Goshima et al., 2008; Meireles et al., 2009; Wainman et al., 2009), showing that Augmin-mediated microtubule nucleation accounts for the assembly of virtually all centrosome-independent spindle microtubules in mitosis.

Surprisingly, a robust bipolar spindle can be formed without the Augmin complex in *Drosophila* oocytes that naturally lack centrosomes (Meireles et al., 2009). This demonstrates that oocytes have an alternative pathway which can assemble spindle microtubules without both Augmin and centrosomes. In stark contrast to the loss of Augmin, removal of Grip71, a component of the γ-tubulin ring complex, strongly reduces spindle microtubules in oocytes (Reschen et al., 2012). This paradoxical result strongly suggests the existence of another nucleation pathway specific to oocytes for assembling a meiotic spindle.

Here we report a novel microtubule nucleation pathway in oocytes, which is mediated by a kinesin-6, Subito/MKlp2. We show that Subito and Augmin recruit Grip71 to the spindle equator and poles, respectively. These two pathways act complementarily to assemble most of the spindle microtubules in oocytes. Furthermore, the N-terminal region of Subito is important to spatially restrict the spindle microtubule nucleation only to the vicinity of meiotic chromosomes. Therefore, this novel nucleation pathway is central for both assembling a meiotic spindle around chromosomes and preventing ectopic microtubule nucleation in the large volume of an oocyte.

## Results

### Grip71/NEDD1 is recruited to the spindle equator in the absence of Augmin in oocytes

In mitotic *Drosophila* cells, few microtubules are assembled in the absence of both centrosomes and Augmin (Goshima et al., 2008; Meireles et al., 2009; Wainman et al., 2009). In contrast, in *Drosophila* oocytes, which naturally lack centrosomes, we have previously shown that spindle microtubules are robustly assembled in the absence of Augmin (Meireles et al., 2009). This difference points to the presence of a yet unknown, oocyte-specific microtubule assembly pathway.

Spindle microtubule assembly in oocytes is greatly reduced in the absence of the γ-tubulin subunit Grip71/NEDD1 (Reschen et al., 2012), suggesting that this new oocyte-specific pathway largely depends on Grip71. Grip71/NEDD1 is a component of the γ-tubulin ring complex through which Augmin recruits the γ-tubulin complex to existing microtubules (Lüders et al., 2006; Vérollet et al., 2006; Zhu et al., 2008; Uehara et al., 2009; Chen et al., 2017). In mitosis, localization of γ-tubulin and Grip71 on the spindle microtubules depends entirely on Augmin (Goshima et al., 2008; Zhu et al., 2008; Wainman et al., 2009). We hypothesized that oocytes have an alternative Augmin-independent pathway which recruits the γ-tubulin complex onto the spindle microtubules through Grip71.

**Figure 1.**
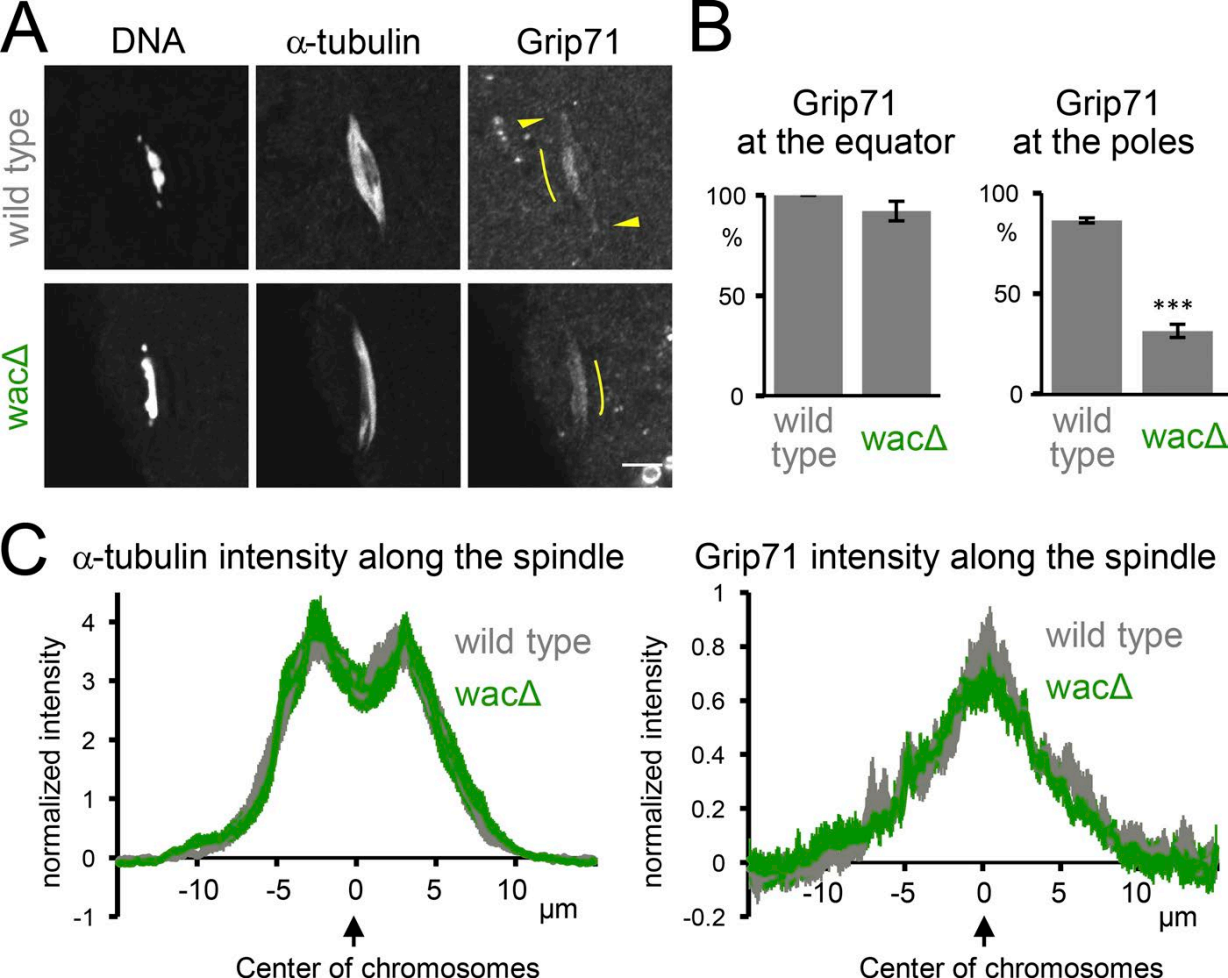
**Grip71 is recruited to the spindle equator independently of Augmin in oocytes. (A)** Immunostaining of a meiotic spindle in mature oocytes from WT and a deletion mutant of *wac* gene encoding an essential subunit of the Augmin complex. Arrowheads indicate Grip71 at the spindle pole, and curved lines indicate the concentration of Grip71 in the spindle equator. Pole localization of Grip71 was greatly reduced in *wac* mutant. Bar, 5 µm. **(B)** The frequencies of meiotic spindles which have Grip71 concentration at the spindle equator and Grip71 localization at least one of the spindle poles, respectively. The graphs show the means (the main bars) and SEM (error bars) from three repeats of experiments for WT and *wacΔ* (38 and 41 spindles in total). *** indicates a significant difference from WT (P < 0.001; *t* test). **(C)** The signal intensities of α-tubulin and Grip71 along the spindle axis in mature oocytes from WT and the *wac* deletion mutant (21 spindles each from three repeats). Three-dimensional image containing each spindle was first projected onto the XY plane, and then projected onto the long axis of the spindle after intense nonspecific Grip71 spots outside of the spindle area were manually removed. The signal intensities were normalized against the background, and the means ± SEM were plotted along the axis after the center of the chromosome mass was aligned.

To test this hypothesis, an antibody was raised against Grip71 (Fig. S1) and used to immunostain mature WT oocytes which naturally arrest in metaphase I. We found that Grip71 is concentrated at the spindle poles and equator (Fig. 1). Next, to test whether this localization depends on Augmin, Grip71 was immunostained in oocytes of a null mutant of the essential Augmin subunit Wac (Meireles et al., 2009). Grip71 localization to the spindle equator was not affected, while Grip71 concentration at the spindle poles appeared to be greatly reduced in *wac* mutant oocytes in comparison with WT (Fig. 1). This Augmin-dependent Grip71 localization to the spindle poles is consistent with our previous observation of Augmin localization to the poles and its function to promote microtubule assembly near the poles in oocytes (Meireles et al., 2009; Colombié et al., 2013). In contrast, the localization of Grip71 to the spindle equator in oocytes is independent of Augmin, which can explain why oocytes robustly assemble spindle microtubules without Augmin, but fail to do so without Grip71 (Meireles et al., 2009; Reschen et al., 2012).

### The *Drosophila* MKlp2 Subito recruits Grip71 to the spindle equator

To understand the molecular mechanism of this novel microtubule assembly pathway, we sought to identify a protein which recruits Grip71 to the spindle equator. We predict that such a protein should (1) colocalize with Grip71 to the spindle equator in oocytes, (2) physically interact with the Grip71/γ-tubulin complex directly or indirectly, and (3) be required for Grip71 localization to the spindle equator but not the spindle poles. We considered Subito (the orthologue of mammalian MKlp2, a kinesin-6) to be a good candidate, as it was previously shown to localize to the spindle equator and have an important role in the integrity of the spindle equator (Jang et al., 2005).

**Figure 2.**
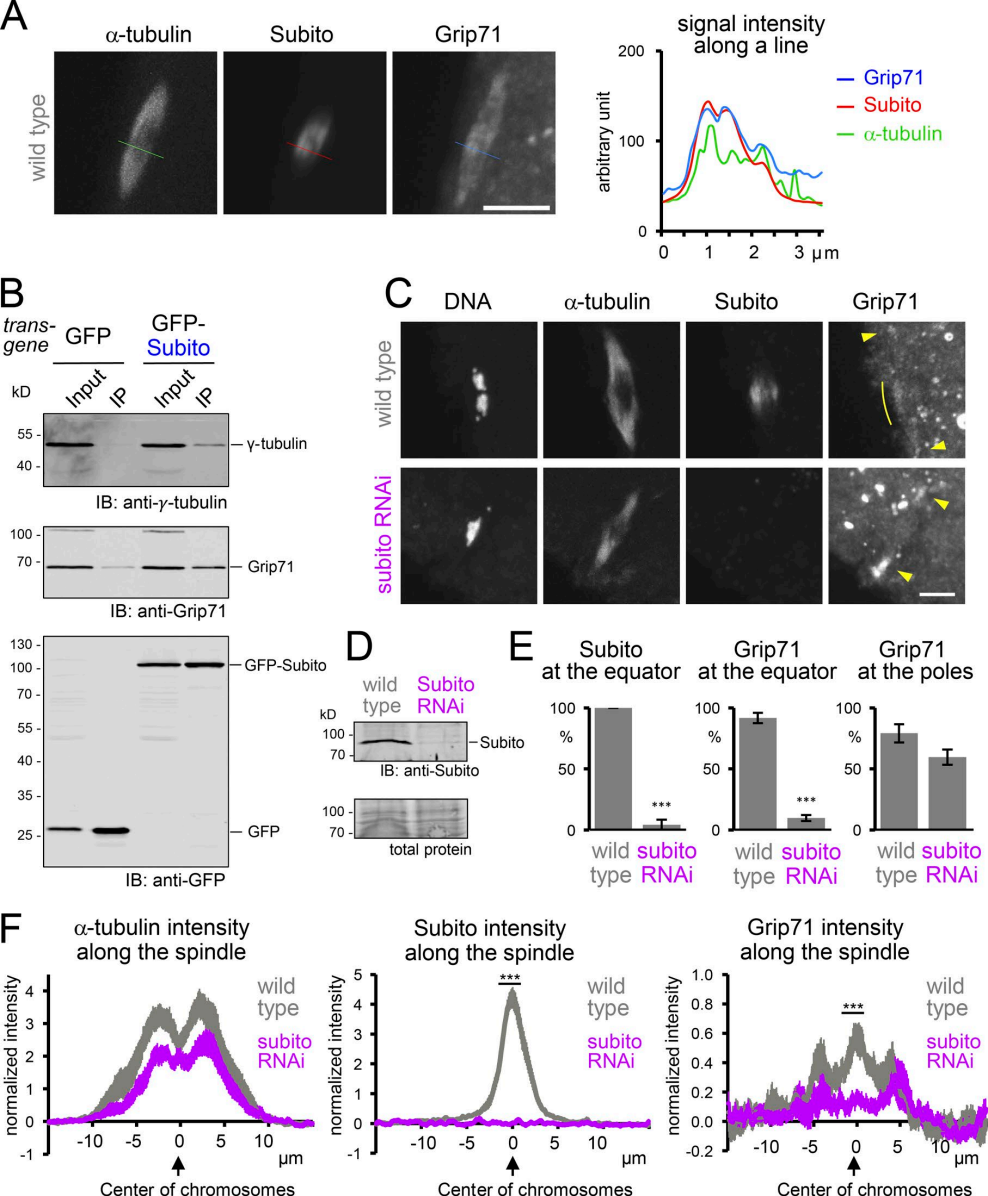
**Subito, the *Drosophila* MKlp2, recruits Grip71 to the spindle equator. (A)** An example of a spindle showing colocalization of Subito with Grip71 in the spindle equator. Immunostaining of a meiotic spindle in mature WT oocytes, and signal intensities (arbitrary unit) of this spindle along the line. Grip71 and Subito are correlated with each other, better than with α-tubulin. Bar, 5 µm. **(B)** Subito physically interacts with γ-tubulin and Grip71. GFP or GFP-Subito was immunoprecipitated from oocytes expressing these proteins in the absence of phosphatase inhibitors, followed by immunoblots (IB). The figure shows one of biological triplicates, all of which showed coimmunoprecipitation. 10% of Input relative to the immunoprecipitated fraction (IP) was loaded. **(C)** Subito is required for Grip71 localization to the spindle equator. Immunostaining of mature WT and *subito* RNAi oocytes. Arrowheads indicate Grip71 localization at the spindle pole, and curved lines indicate Grip71 concentration in the spindle equator. The equator localization of Grip71 was lost in the *subito* RNAi oocyte. Bar, 5 µm. **(D)** Immunoblot of ovaries from WT and *subito* RNAi flies. **(E)** The frequencies of meiotic spindles which have Subito and Grip71 concentration at the spindle equator and Grip71 localization at at least one of the spindle poles, respectively. The graphs show the means and SEM from three repeats for each of WT and *subito* RNAi (48 and 61 spindles in total). *** indicates significant differences from WT (P < 0.001; *t* test). **(F)** The signal intensities of α-tubulin, Subito and Grip71 along the spindle axis in mature oocytes from WT and *subito* RNAi (14 spindles each from two repeats), plotted in the same way as Figure 1 C. *** indicates significant differences (P < 0.001; *t* test) in the mean signal intensity of the equator region (the region between −1 and +1 µm).

To test whether Subito colocalizes with Grip71 to the spindle equator, mature WT oocytes were coimmunostained for Subito, Grip71, and α-tubulin. Subito and Grip71 showed nearly identical localization patterns in the spindle equator, while α-tubulin showed a distinct pattern from them (Fig. 2 A). This was confirmed by intensity measurements along a cross section of a spindle (Fig. 2 A). To test physical interaction between Subito and Grip71, GFP-Subito expressed in ovaries was immunoprecipitated for analysis by Western blot. Grip71 and γ-tubulin were both coimmunoprecipitated with GFP-Subito from a *Drosophila* ovary extract (Fig. 2 B), demonstrating that Subito interacts with Grip71 and γ-tubulin.

To test whether Grip71 localization depends on Subito, mature oocytes expressing shRNA against *subito* were immunostained for Subito, Grip71, and α-tubulin (Fig. 2 C). Subito signals were dramatically reduced, confirming the specificity of the anti-Subito antibody and the effectiveness of RNAi (Fig. 2, C–F). Strikingly, Grip71 localization to the spindle equator was lost (Fig. 2, C, E, and F). In contrast, Grip71 localization at the poles was still observed, although in fewer oocytes (Fig. 2, C and E). Immunoblots showed that Grip71 depletion reduced the amount of Subito in ovaries. Moreover, Subito depletion also reduced the amount of Grip71, but only to a degree which does not account for the dramatic loss of Grip71 at the spindle equator in Subito-depleted oocytes (Fig. S2). Therefore, Subito is essential for Grip71 localization to the spindle equator, but largely dispensable for its localization to the poles. Collectively, we conclude that Subito recruits Grip71 to the spindle equator in oocytes.

### Subito and Augmin complement each other to generate spindle microtubules

**Figure 3.**
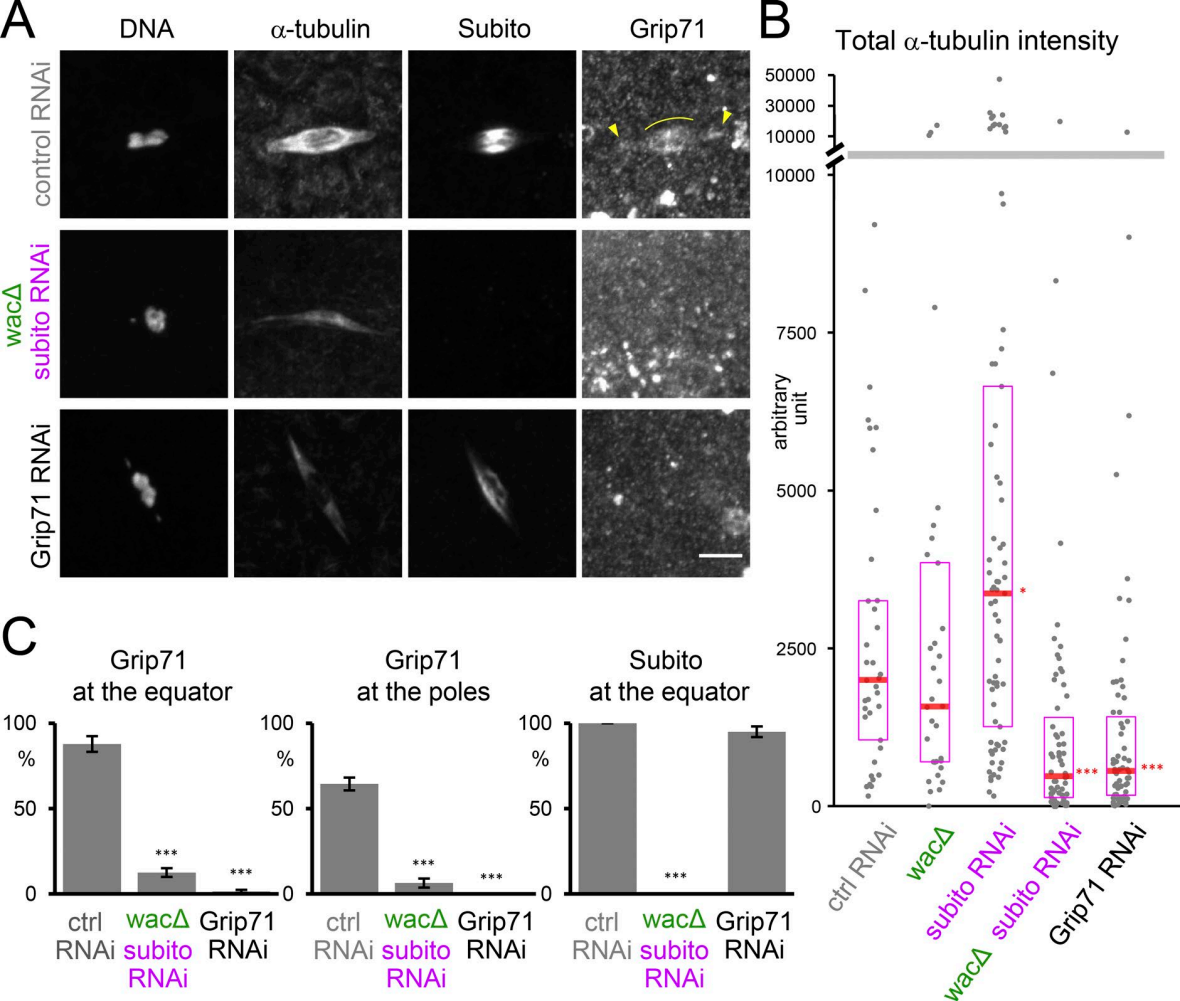
**Subito and Augmin complement each other to generate microtubules. (A)** Immunostaining of a meiotic spindle in mature oocytes from WT expressing control (*white*) shRNA, a *wac* deletion mutant expressing *subito* shRNA, and WT expressing *Grip71* shRNA. Arrowheads indicate Grip71 localization at the spindle pole, and curved lines indicate Grip71 concentration on the spindle equator. Spindle microtubules were greatly reduced and Grip71 localization on the spindle was lost in the *wac* deletion mutant expressing *subito* shRNA. These images were taken using the same settings and are shown without contrast enhancement. Bar, 5 µm. **(B)** The total tubulin intensity of the meiotic spindle in each genotype (37, 33, 73, 63, and 67 spindles). RNAi of the *white* gene was used as the control RNAi. The median is indicated by the central line, and the second and third quartiles are indicated by the box. The y axis is broken into two with different scales to show all data. Data were pooled from triplicate experiments. **(C)** The frequencies of meiotic spindles which have Grip71 concentration at the spindle equator and Grip71 localization at at least one of the spindle poles, respectively, and Subito at the equator. The graphs show the means and SEM from four repeats for each genotype (53, 89, and 98 spindles in total). * and *** indicate significant differences from control RNAi (P < 0.05 and 0.001, respectively; Wilcoxon rank-sum test for B*, t* test for C).

It was previously shown that the total amount of spindle microtubules was not significantly reduced in a null mutant of either Subito or the Augmin subunit Wac in oocytes (Giunta et al., 2002; Jang et al., 2005; Colombié et al., 2008; Meireles et al., 2009). We hypothesized that these two pathways may act complementarily to assemble spindle microtubules. To test this, both Subito and Wac were simultaneously depleted from oocytes by expressing shRNA against Subito in ovaries of the *wac* null mutant. Immunostaining showed that a single depletion of Subito or Wac did not reduce the total amount of spindle microtubules (Fig. 3 B), as previously reported (Giunta et al., 2002; Jang et al., 2005; Colombié et al., 2008; Meireles et al., 2009). In contrast, simultaneous depletion of Subito and Wac led to a dramatic reduction of spindle microtubules and often thinner spindles (Fig. 3, A and B). This phenotype is reminiscent of the *grip71* RNAi or null mutant phenotype (Fig. 3, A and B; Reschen et al., 2012), suggesting that Grip71 recruitment to the meiotic spindle depends entirely on Subito and Wac. Indeed, immunostaining showed that Grip71 fails to localize to the spindle in oocytes codepleted of Subito and Wac (Fig. 3, A and C). Remaining microtubules in the absence of Grip71, or simultaneous absence of Subito and Wac, are consistent with the previous reports indicating that not all microtubule nucleation depends on the γ-tubulin ring complex (Colombié et al., 2006; Wiese and Zheng, 2006). Therefore, we conclude that Subito and Augmin mediate two complementary pathways which recruit Grip71/γ-tubulin to assemble most spindle microtubules in oocytes.

### Grip71 mediates ectopic microtubule assembly induced by Subito lacking the N-terminal region

**Figure 4.**
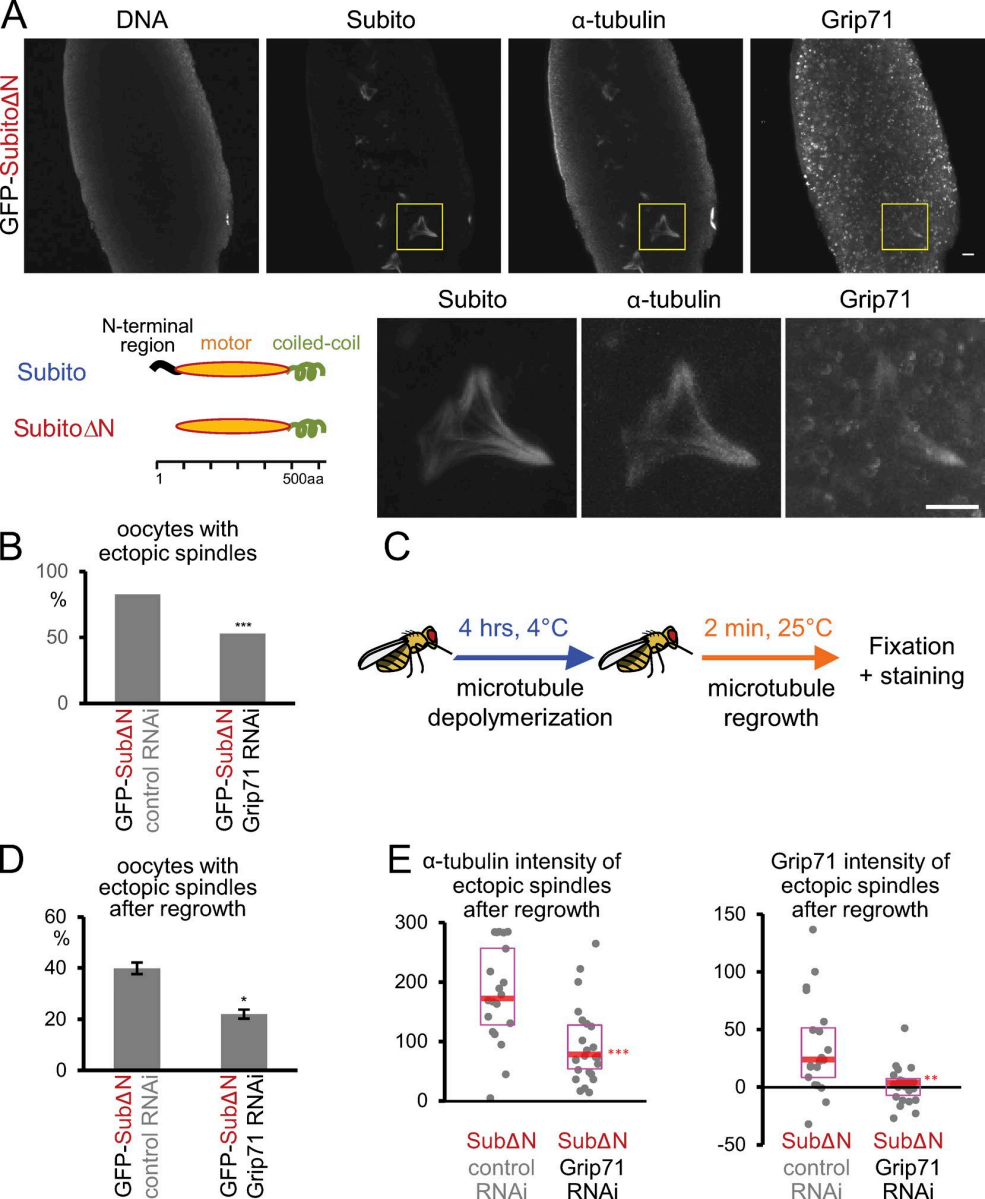
**Grip71 mediates ectopic microtubule assembly induced by expression of Subito lacking the N-terminal region. (A)** Grip71 is recruited to the ectopic spindles in oocytes expressing Subito lacking the N-terminal 89 residues (SubitoΔN). Immunostaining of mature WT oocytes expressing GFP-SubitoΔN that lacks the N-terminal nonmotor domain as shown in the diagram. GFP-SubitoΔN was detected by an antibody against Subito. The boxed area is magnified below. Bars, 10 µm. **(B)** The frequencies of oocytes which have ectopic spindles when GFP-SubitoΔN was expressed together with control (*white*) or Grip71 shRNA. 100 and 160 oocytes from >24 flies were examined, respectively. *** indicates a significant difference from WT (P < 0.001; Fischer's exact test). **(C)** Schematic diagram of microtubule regrowth experiments. **(D)** The frequencies of oocytes with ectopic spindles when GFP-SubitoΔN was expressed together with shRNA against control (*white*) or *Grip71* after microtubule regrowth. The graph shows the means and SEM from two repeats for each genotype (202 and 277 oocytes in total). * indicates a significant difference from WT (P < 0.05; *t* test). **(E)** Signal intensities of α-tubulin and Grip71 on ectopic spindles above the cytoplasmic background after microtubule regrowth in each genotype (20 and 24 oocytes). The graphs show the data from one of two repeated experiments, both of which showed decreases to similar levels in Grip71 RNAi oocytes. The median is indicated by the central line, and the second and third quartiles are indicated by the box. ** and *** indicates a significant difference from control RNAi (P < 0.01 and 0.001, respectively; Wilcoxon rank-sum test).

It was previously shown that overexpression of Subito lacking its N-terminal nonmotor region in oocytes can induce ectopic spindles in the cytoplasm, away from the chromosomes (Jang et al., 2007; Fig. 4 A). Originally it was thought that mis-regulated Subito bundles cytoplasmic microtubules, resulting in the formation of ectopic spindles (Jang et al., 2007). However, an alternative interpretation is that mis-regulated Subito ectopically recruits the γ-tubulin complex in the ooplasm and nucleates microtubules, independently of chromosomes. If this interpretation is correct, we predict that (1) Grip71 is recruited to the ectopic sites of microtubule assembly and that (2) the formation of the ectopic spindles depends on Grip71.

To test whether Grip71 is recruited to ectopic spindles, we immunostained Grip71 in oocytes expressing Subito lacking the N-terminal region (SubitoΔN). As previously shown (Jang et al., 2007), SubitoΔN not only localized to the equatorial region of the meiotic spindle associated with chromosomes, but was also concentrated on ectopic spindles in oocytes (Fig. 4 A). Importantly, Grip71 was recruited to ectopic spindles in addition to the equatorial region of the meiotic spindle (Fig. 4 A).

Next, to determine whether the formation of the ectopic spindles depends on Grip71, Grip71 was depleted by RNAi from oocytes expressing SubitoΔN (Fig. 4 B). >80% of oocytes expressing SubitoΔN and control shRNA form ectopic spindles. In contrast, when Grip71 is depleted, about a half of the oocytes expressing SubitoΔN failed to form ectopic spindles. To assess microtubule nucleation more accurately, microtubule regrowth experiments were performed using cold treatment followed by warming (Fig. 4 C). Cold treatment abolished both chromosome-associated spindles and ectopic microtubules. 2 min after warming, ∼40% of control oocytes displayed ectopic microtubule arrays associated with Grip71 foci, while fewer Grip71-depleted oocytes (∼20%) displayed ectopic arrays (Fig. 4 D). Furthermore, these fewer ectopic arrays formed in Grip71-depleted oocytes showed substantially lower tubulin intensity than the control (Fig. 4 E). Fewer and weaker microtubule arrays in Grip71-depleted oocytes indicates that ectopic microtubule assembly by SubitoΔN is largely dependent on Grip71. These results support our hypothesis that SubitoΔN induces ectopic microtubule nucleation through recruiting the γ-tubulin complex in the ooplasm.

### Subito and its associated proteins can induce microtubule nucleation in vitro

**Figure 5.**
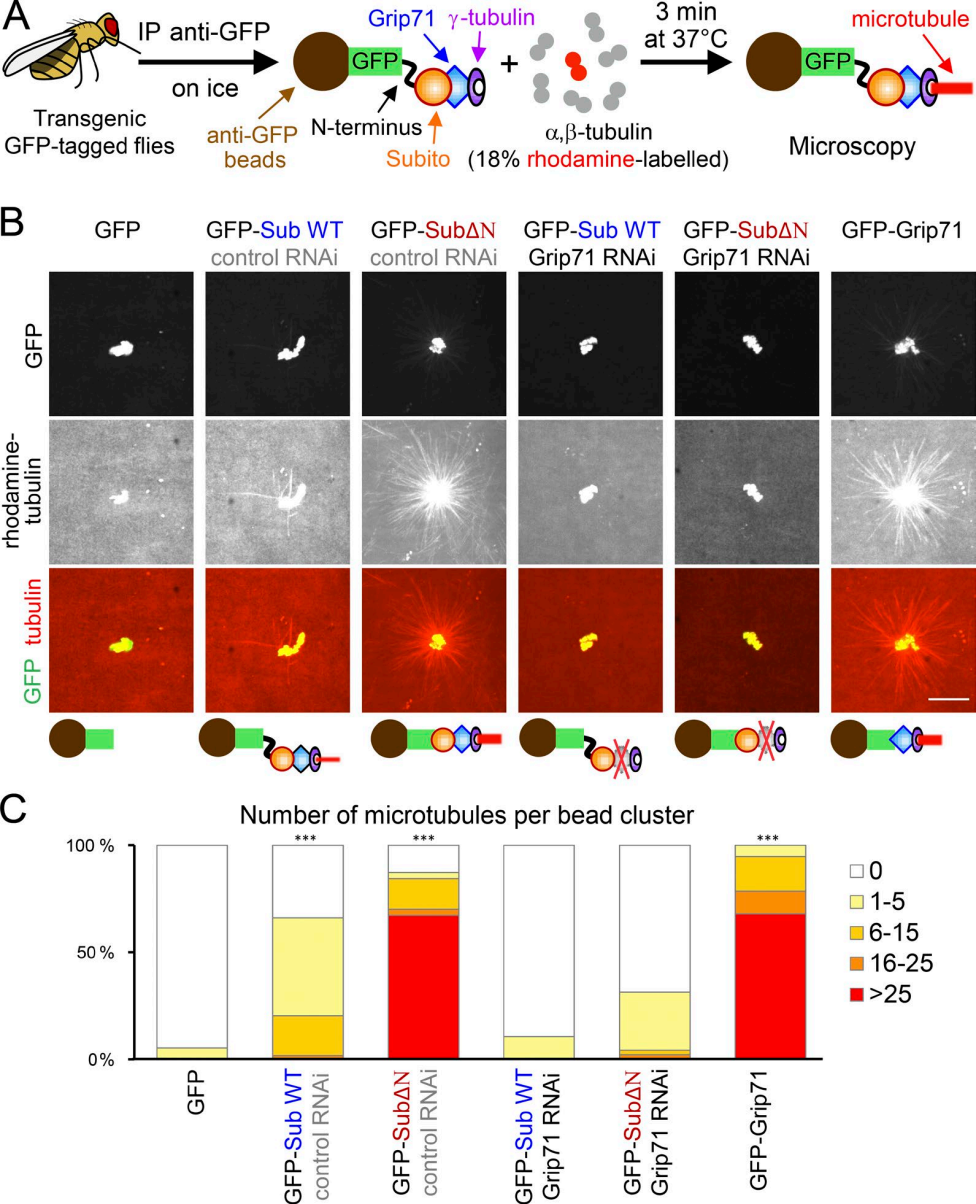
**The Subito complex from ovaries can induce microtubule nucleation in vitro. (A)** Schematic diagram of the assay of in vitro microtubule nucleation activity using an immunoprecipitated GFP-tagged protein (such as GFP-Subito) on beads incubated with pure pig α,β-tubulin dimer. **(B)** GFP-tagged protein was immunoprecipitated from oocytes expressing the protein alone or together with control (*white*) or *Grip71* shRNA. These images were taken using the same settings and the same alteration of the brightness and contrast was applied to all to show beads and microtubule asters clearly. Bar, 10 µm. **(C)** The number of microtubules per bead cluster was counted for each genotype (75, 59, 70, 76, 48, and 56 bead clusters). *** indicates a significant difference from GFP beads in the proportion of bead clusters with ≥6 microtubules (P < 0.001; Fisher's exact test). The graph shows the data from one of two repeated experiments, both of which showed similar results. In both experiments, GFP-SubitoΔN and GFP-Grip71 beads have a high microtubule nucleation activity, while GFP-Subito (GFP-Sub WT) beads have a lower nucleation activity. These activities depend on Grip71. GFP beads have virtually no nucleation activity.

Our genetic and cytological studies support the hypothesis that Subito recruits γ-tubulin through Grip71 to nucleate spindle microtubules. To biochemically demonstrate that Subito and its associated proteins can nucleate microtubules, we set up the following in vitro assay using GFP-tagged Subito immunoprecipitated from ovaries incubated with pure pig brain α,β-tubulin dimer (Fig. 5 A).

Ovaries of flies expressing GFP-Subito (Subito WT) or GFP-SubitoΔN were used to immunoprecipitate each Subito variant using beads coupled with an anti-GFP antibody. In addition to GFP-tagged proteins, those oocytes also expressed shRNA against either *Grip71* or *white* (control). As negative and positive controls, we used GFP alone or GFP-Grip71 immunoprecipitated from corresponding ovaries. These beads were then incubated with pure α,β-tubulin dimer (18% of which were rhodamine labeled) and GTP. After 3 min at 37°C, the reaction was fixed and observed under a fluorescence microscope.

As expected, the GFP-beads were associated with few microtubules, while the GFP-Grip71 beads were associated with an aster-like microtubule array (Fig. 5, B and C). Strikingly, GFP-SubitoΔN beads from control RNAi oocytes were associated with an aster-like microtubule array to a similar extent to GFP-Grip71 beads (Fig. 5, B and C). Interestingly, GFP-Subito WT beads from control RNAi oocytes were also associated with some microtubules, but to a much lesser extent than GFP-SubitoΔN beads (Fig. 5, B and C). This stronger in vitro nucleation activity of GFP-SubitoΔN is consistent with its ability to induce ectopic microtubules in vivo.

It is formally possible that microtubules around the GFP-SubitoΔN beads were spontaneously nucleated in the solution and then captured by the beads, rather than nucleated by the beads. To exclude this possibility, we counted the number of free microtubules not associated with beads (Fig. S3). If microtubules were spontaneously nucleated and then captured, fewer free microtubules should be observed in the experiments using GFP-SubitoΔN beads than with GFP-beads. In contrast, if GFP-SubitoΔN beads nucleated microtubules (some of which were detached), more free microtubules should be observed than GFP-beads. Indeed, we observed many more free microtubules in the experiment using GFP-SubitoΔN beads than using GFP-beads (Fig. S3). This confirmed that GFP-SubitoΔN beads induced microtubule nucleation, rather than simply capturing spontaneously nucleated microtubules.

To further confirm that these microtubule asters formed around the GFP-SubitoΔN or GFP-Subito WT beads were due to microtubule nucleation, we tested whether a component of the γ-tubulin ring complex, Grip71, is required for aster formation. GFP-SubitoΔN or GFP-Subito WT was immunoprecipitated from ovaries simultaneously expressing GFP-SubitoΔN or GFP-Subito WT, and shRNA against Grip71. None or a few microtubules were assembled around the GFP-SubitoΔN or GFP-Subito WT beads from Grip71-depleted oocytes (Fig. 5, B and C). These results demonstrate that microtubule assembly mediated by the GFP-SubitoΔN or GFP-Subito WT beads depends on Grip71. Therefore, we conclude that Subito and its associated proteins can nucleate microtubules from pure α,β-tubulin in vitro, and that this nucleation activity is inhibited by the N-terminal region of Subito.

### Subito interaction with the γ-tubulin complex is suppressed by its N-terminal region

**Figure 6.**
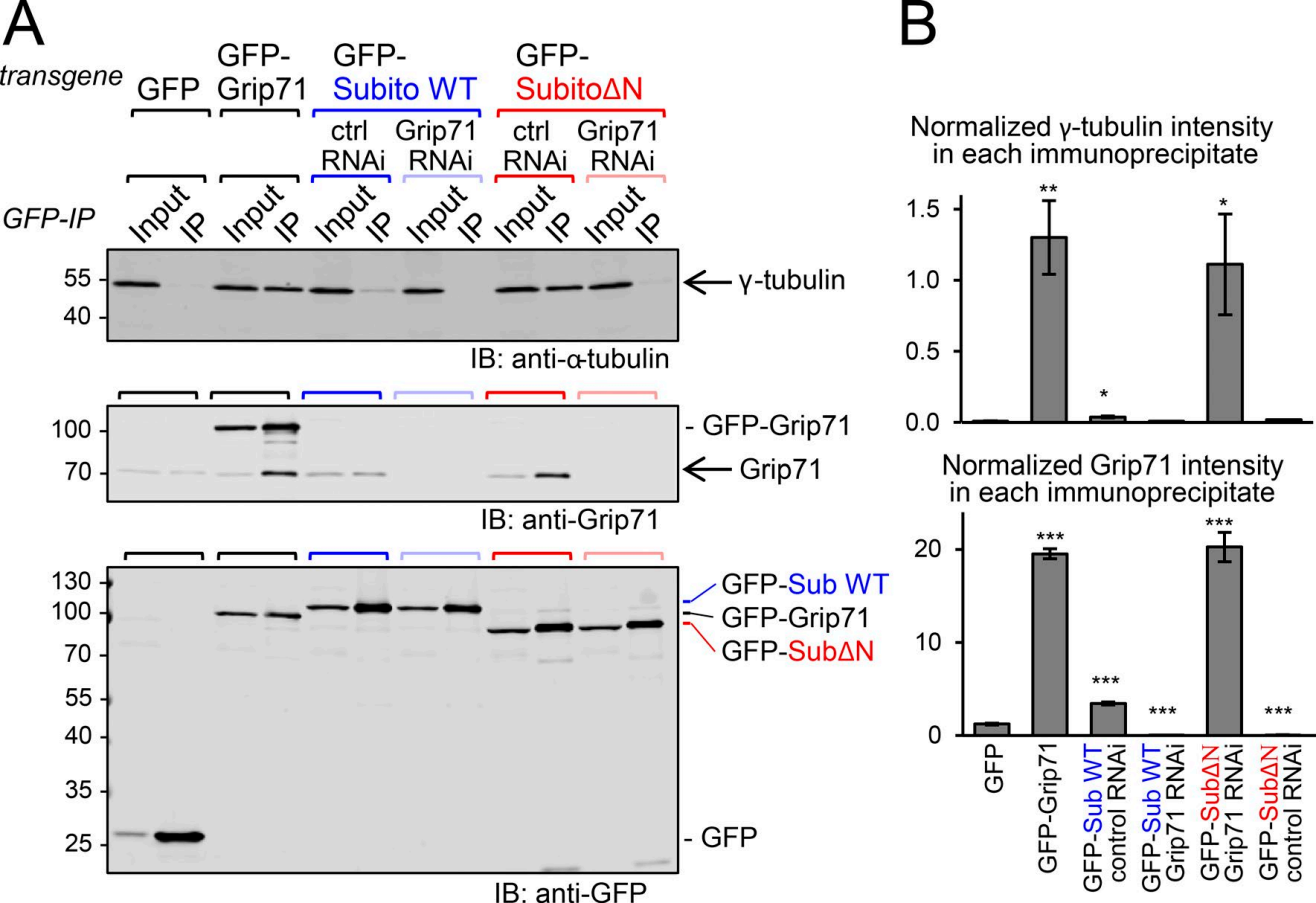
**Subito interaction with Grip71 is negatively regulated by its N-terminal region. (A)** GFP-tagged protein was immunoprecipitated from oocytes expressing the protein alone or together with control (*white*) or *Grip71* shRNA in the presence of phosphatase inhibitors, followed by immunoblots (IB). 2.5% of Input relative to the immunoprecipitated fraction (IP) was loaded. γ-Tubulin and Grip71 were efficiently coimmunoprecipitated with GFP-SubitoΔN, but only weakly with GFP-Subito. Coimmunoprecipitation of γ-tubulin depends on Grip71. This figure shows the immunoblot from one of three repeated experiments, all of which provided similar results. **(B)** Normalized signal intensity of γ-tubulin and Grip71 in each immunoprecipitate. The signal intensity of the IP lane was normalized using the γ-tubulin intensity in the Input lane in each immunoprecipitation. The means and SEM from triplicate experiments were shown. *, **, and *** indicate significant differences from GFP (P < 0.05, P < 0.01, and P < 0.001, respectively; *t* test).

Genetic and cytological analysis in this study showed that the Subito N-terminal region suppresses the Subito activity and prevents ectopic microtubule nucleation in oocytes. Furthermore, the N-terminal region suppresses in vitro microtubule nucleation activity of Subito. To gain a mechanistic insight, we tested whether the interaction between Subito and the γ-tubulin complex is regulated by the N-terminal region. Proteins coimmunoprecipitated with GFP-Subito or GFP-SubitoΔN were analyzed by immunoblot using Grip71 and γ-tubulin antibodies (Fig. 6).

Strikingly, we found that Grip71 and γ-tubulin were coimmunoprecipitated with GFP-SubitoΔN much more efficiently than with GFP-Subito (Fig. 6). This demonstrated that the N-terminal region suppresses the interaction between Subito and the γ-tubulin complex, which explains why SubitoΔN is hyperactive in nucleating microtubules in vivo and in vitro. When Grip71 was depleted from ovaries, γ-tubulin was not coimmunoprecipitated with either GFP-SubitoΔN or Subito, confirming that Grip71 mediates the interaction between Subito and the γ-tubulin complex (Fig. 6). These results show that the N-terminal region inhibits the interaction between Subito and Grip71, hence between Subito and the γ-tubulin complex.

## Discussion

Our in vivo and in vitro analysis uncovered a novel microtubule nucleation pathway specific to oocytes, which is mediated by the conserved kinesin-6 Subito/MKlp2. Subito recruits the γ-tubulin complex to the spindle equator via Grip71/NEDD1, acting complementarily to Augmin which fulfils this role at the spindle poles. A loss of either pathway does not reduce bulk spindle microtubules, but together, they are essential for the assembly of most spindle microtubules in oocytes. We demonstrated that Subito can interact with the γ-tubulin complex through Grip71 and induce microtubule nucleation from pure tubulin dimers in vitro. Interestingly the N-terminal region of Subito suppresses its interaction with the γ-tubulin complex, and prevents ectopic assembly of microtubules in oocytes. Therefore, this novel microtubule nucleation pathway is central to assemble the meiotic spindle around chromosomes and, as importantly, to suppress ectopic microtubule nucleation in the ooplasm.

**Figure 7.**
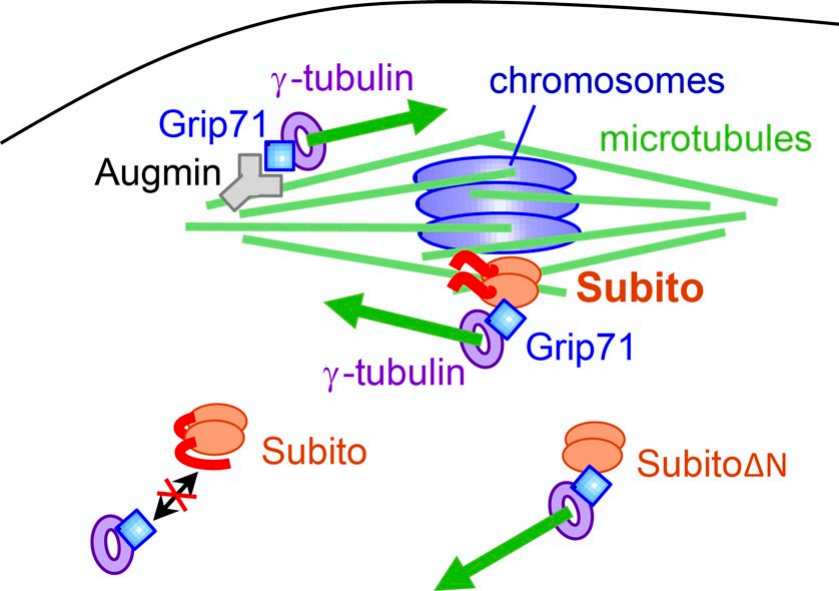
**Subito recruits the γ-tubulin complex to nucleate microtubules in the spindle equator in oocytes.** Schematic model of meiotic spindle assembly in oocytes. Subito and Augmin recruit the γ-tubulin complex to the spindle equator and poles, respectively, through Grip71. This nucleates a new microtubule to amplify spindle microtubules. These two pathways work complementarily to assemble most of the spindle microtubules in oocytes. The N-terminal region of Subito suppresses the interaction with the γ-tubulin complex away from chromosomes, but this suppression is removed near meiotic chromosomes. Subito lacking the N-terminal region constitutively associates with the γ-tubulin complex and induces ectopic microtubule nucleation away from chromosomes in the ooplasm.

We propose the following model of spatially restricted spindle assembly in *Drosophila* oocytes (Fig. 7). In the vicinity of the chromosomes, Subito binds to microtubules in the spindle equator and recruits the γ-tubulin complex through Grip71 to nucleate new microtubules. In the same manner, Augmin localizes to the spindle poles where it nucleates microtubules. Away from chromosomes, the N-terminal region of Subito suppresses its interaction with the γ-tubulin complex and therefore prevents ectopic microtubule nucleation.

Taking advantage of *Drosophila* oocytes, we combined genetic, cytological, and biochemical approaches to uncover a novel oocyte-specific microtubule nucleation pathway. Unique features of oocytes, such as lack of centrosomes and a large volume, are also characteristics of oocytes from other species including humans. Furthermore, all proteins mentioned here, such as Subito/MKlp2, Grip71/NEDD1, Augmin, and the γ-tubulin complex, are widely conserved (Jang et al., 2005; Haren et al., 2006; Lüders et al., 2006; Lawo et al., 2009; Uehara et al., 2009; Kollman et al., 2011; Teixidó-Travesa et al., 2012). Therefore, a similar mechanism to this novel nucleation pathway is likely to be operating in oocytes of other species to assemble the meiotic acentrosomal spindle.

Subito/MKlp2 belongs to a family of conserved plus end directed microtubule motors with microtubule bundling activity (Neef et al., 2003). Examples of kinesins interacting with and regulating the γ-tubulin complex have been reported previously (Olmsted et al., 2013, 2014). In addition, our study on Subito provides the first biochemical evidence of a kinesin recruiting an active γ-tubulin complex. By combining microtubule binding and nucleation activities, Subito could locally increase the number of microtubules in an exponential manner to trigger rapid assembly of a very dense microtubule network (which is called microtubule amplification; Zhu et al., 2008; Goshima and Kimura, 2010). Augmin was the only previously known factor which mediates microtubule amplification for spindle assembly (Meunier and Vernos, 2016; Prosser and Pelletier, 2017). In oocytes, we showed that the Subito pathway acts complementarily to Augmin pathway to assemble most of the spindle microtubules in oocytes. In mitosis, however, Subito microtubule nucleation pathway seems to be inactive or negligible, as very few microtubules are assembled in the absence of both centrosomes and Augmin (Goshima et al., 2008; Meireles et al., 2009; Wainman et al., 2009). Moreover, Subito depletion does not result in significant spindle defect in mitotic cells lacking centrosomes (Moutinho-Pereira et al., 2013). Therefore, this new role of Subito in microtubule nucleation appears to be specific to oocytes. Nevertheless, it is still a possibility that Subito/MKlp2 and/or Pavarotti/MKlp1 may be involved in microtubule nucleation to form the central spindle during mitotic anaphase/telophase.

During animal mitosis, centrosomes are major microtubule nucleating sites (Prosser and Pelletier, 2017) and Augmin nucleates randomly along the spindle (Goshima et al., 2008). Based on our findings, we hypothesize that the absence of the centrosomal activity in oocytes is compensated by two oocyte-specific adaptations of Augmin and Subito. First, oocyte-specific localization of Augmin at the spindle poles compensates for the absence of nucleation activity of centrosomes from the poles (Meireles et al., 2009; Colombié et al., 2013). Second, oocyte-specific Subito pathway compensates for the absence of microtubule nucleation by Augmin in the equator region.

Once microtubules are nucleated in the spindle equator, they are cross-linked, and their polarity is sorted out by microtubule motors regardless of their original polarity (Bennabi et al., 2016). As the mammalian orthologue of Subito, MKlp2, has been shown to bundle microtubules (Neef et al., 2003), Subito can potentially coordinate nucleation, cross-linking and polarity sorting during meiotic spindle assembly.

In addition, our study highlights a central role of Subito in spatially restricting microtubule amplification to the vicinity of chromosomes in *Drosophila* oocytes. This spatial regulation is crucial, as oocytes commonly contain exceptionally large cytoplasmic volumes. We showed that the N-terminal nonmotor region of Subito suppresses ectopic microtubule nucleation by inhibiting the interaction with Grip71, the subunit responsible for recruiting the γ-tubulin complex. It is possible that the N-terminal region may recruit a protein which inhibits the interaction with Grip71. Alternatively, there are many examples of auto-inhibition of kinesins by their nonmotor region (Verhey and Hammond, 2009). It is therefore likely that a signal from chromosomes locally abolishes auto-inhibition of the Subito nucleation activity to trigger spindle microtubule nucleation in a spatially confined manner. In addition, a chromosomal signal may locally promote the microtubule binding and equator localization of Subito. In simple terms, chromosomes switch on Subito-dependent nucleation, while this pathway remains switched off in the cytoplasm of the oocyte. Collectively, tight regulation of Subito appears critical to assemble spindle microtubules around the chromosomes and to prevent their assembly anywhere else.

Ran-GTP or Aurora B activity could potentially act as the signal from the meiotic chromosomes to remove auto-inhibition of Subito (Bennabi et al., 2016; Radford et al., 2017). We identified the nuclear localization signal (NLS; the binding site of importin regulated by Ran) in the N-terminal region. The NLS is required to localize Subito in the nucleus in interphase cells, but it is not required for suppression of the Subito activity in oocytes (Fig. S4). Recent evidence suggests that serine residues in the N-terminal region are important for restricting Subito activity and localization (Das et al., 2018). It would be of great future interest to elucidate the regulatory mechanism of Subito in oocytes.

## Materials and methods

### Recombinant DNA techniques

Standard molecular techniques (Sambrook et al., 1989) were followed throughout. cDNA of each gene of interest was cloned first into pENTR/D-TOPO using pENTR Directional Topo Cloning kit (Invitrogen). The absence of unwanted mutations was confirmed by DNA sequencing using BigDye (PerkinElmer). cDNAs were then recombined into Gateway destination vectors using LR Clonase II reaction following the manufacturer’s protocol (Invitrogen). To generate transgenic flies expressing a GFP-tagged protein under the UASp promoter, we used ϕPGW modified from destination vector pPGW of the Murphy’s Gateway collection by adding the ϕC31 attB recombination site (Beaven et al., 2017). To generate S2 cells expressing GFP-tagged protein under the Cu^2+^-inducible metallothionein promoter, we used pMTGW (a gift from G. Goshima, Nagoya University, Japan), a Gateway vector derived from pMT (Invitrogen).

To generate the *Grip71* entry clone, an entire coding region was amplified from cDNA (RE05579) by PCR. This cDNA contains a cytosine deletion at 2,134 bp that induces a premature stop codon. The cytosine has been added back to restore Grip71 complete coding region (1–646 amino acids followed by a stop codon) using the Quick Change II XL Site-Directed Mutagenesis kit (Promega) and the following primers: 5′-CCAACATTTCCGTGGCCAGCAGCACAGGAGGCGGCAGCG-3′ and 5′-CGCTGCCGCCTCCTGTGCTGCTGGCCACGGAAATGTTGG-3′.

To generate the *subito* entry clone, a full-length coding region (encoding 1–628 amino acids followed by a stop codon) or a part of the coding region (SubitoΔN encoding 90–628 amino acids followed by a stop codon) was amplified from cDNA (LD35138) by PCR.

To generate Subito NLS*, a putative NLS, RPRPNKKMRLF (30th–40th residues), was identified using cNLS mapper (Kosugi et al., 2009) with a maximum cut-off score of 7. Arginine 32, lysine 35, lysine 36 and arginine 38 were sequentially mutated into alanine in the *subito* entry plasmid using the Quick Change II XL Site-Directed Mutagenesis kit (Promega) and the following primers, respectively: 5′-CCGCCGCTTTCGACCGGCACCCAACAAAAAGATG and 5′-CATCTTTTTGTTGGGTGCCGGTCGAAAGCGGCGG-3′; 5′-CCGCTTTCGACCGGCACCCAACGCAAAGATGCGTCTGTTTGACAACAT-3′ and 5′-ATGTTGTCAAACAGACGCATCTTTGCGTTGGGTGCCGGTCGAAAGCGG-3′; 5′-CGCTTTCGACCGGCACCCAACGCAGCGATGCGTCTGTTTGACAACAT-3′ and 5′-ATGTTGTCAAACAGACGCATCGCTGCGTTGGGTGCCGGTCGAAAGCG-3′; 5′-GGCACCCAACGCAGCGATGGCTCTGTTTGACAACATTCAG-3′ and 5′-CTGAATGTTGTCAAACAGAGCCATCGCTGCGTTGGGTGCC-3′.

For flies expressing *Grip71* shRNA, a Walium22 plasmid carrying shRNA against position 63 of the 5′UTR of the *Grip71* gene was generated following TRiP protocol (http://hwpi.harvard.edu/files/fly/files/2ndgenprotocol.pdf?m=1465918000; accessed April 8, 2018) using the following primers: 5′-CTAGCAGTGTGTAAATATCTGAAGAAATATAGTTATATTCAAGCATATATTTCTTCAGATATTTACACGCG-3′ and 5′-AATTCGCGTGTAAATATCTGAAGAAATATATGCTTGAATATAACTATATTTCTTCAGATATTTACACACTG-3′.

For Fig. 4 B, an additional line expressing a different shRNA directed against position 375 of the coding region of *Grip71* gene was generated using the following primers: 5′-CTAGCAGTGAGCGGTTGTGTTAAGCTATATAGTTATATTCAAGCATATATAGCTTAACACAACCGCTCGCG-3′ and 5′-AATTCGCGAGCGGTTGTGTTAAGCTATATATGCTTGAATATAACTATATAGCTTAACACAACCGCTCACTG-3′.

For RNAi in S2 cells, dsRNA against the β-lactamase gene was used as a control as previously described (Syred et al., 2013). For *Grip71* RNAi in S2 cells (Fig. S1, A and B), double-stranded RNAs were amplified in the first PCR using the following pairs of primers containing half of the T7 promoter and the full T7 promoter sequence was added in the second PCR using the primer, 5′-CGACTCACTATAGGGAGAGCGGGGGATTCTCTTTGT-3′. In the first PCR, the region 504–925 bp of Grip71 coding sequence was amplified using 5′-CGACTCACTATAGGGAGACCGAGCAAACGCTTTCAT-3′ and 5′-CGACTCACTATAGGGAGATGCTGGTCATCCCCACTT-3′ for Grip71 RNAi 1, and the region 1,062–1,479 bp of Grip71 coding sequence was amplified by 5′-CGACTCACTATAGGGAGATTGGTTACGGGGTGTCAA-3′ and 5′-GAATTAATACGACTCACTATAGGGAGA-3′ for Grip71 RNAi 2. The PCR products were in vitro transcribed and purified using MEGAscript T7 High Yield Transcription kit (Ambion) following the manufacturer’s instructions.

### *Drosophila* techniques

Standard methods of fly handling (Ashburner et al., 2005) were used. *w^1118^* was used as WT in this study. For a *Grip71* deletion mutant, we used *Dgp71WD^120^* (FBal0281724) over a deficiency from a cross between *Dgp71WD^120^/SM6A* (Reschen et al., 2012) and a deficiency *Df(2L)ED1196/SM6A* (FBab0031555). The *wac* deletion mutant line used in this study was a homozygote from a *wac^Δ12^/TM6C* stock (FBal0220993; Meireles et al., 2009). A line expressing shRNA against *subito* (GL00583/FBti0146499) was used to inhibit *subito* expression. Two lines expressing shRNA against *Grip71* 5′UTR or the coding region (this study) were used to inhibit *Grip71* expression. In Fig. 4 B, as both lines gave very similar results, they were pooled together for the analysis. For other experiments, the line expressing shRNA against *Grip71* 5′UTR was used. The line (GL00094/FBti0144194) expressing shRNA against *white* was used as a negative control (control RNAi) in this study. shRNA and transgenes encoding for GFP-Grip71, GFP-Subito WT, and GFP-SubitoΔN under the control of the UASp promoter were expressed using one copy of a female germline specific GAL4 driver, *nos-GAL4-VP16 MVD1* (FBti0012410), except Fig. 2. In Fig. 2, a maternal-tubulin GAL4 driver V37 (FBti0016914) was used in one of the three experiments, but as it gave a similar result to the other two using MVD1, all three experiments were analyzed together. To express GFP-Subito WT or GFP-SubitoΔN together with *white* shRNA (a negative control) or *Grip71* shRNA, a recombinant chromosome between two transgenes (both on the third chromosome) was generated and then crossed with MVD1. Recombinant chromosomes were established by selecting and confirming using visible markers and PCR after meiotic recombination in females.

To generate transgenic fly lines, ϕPGW carrying cDNA encoding the full-length Subito (Subito WT), SubitoΔN, Subito NLS* and an empty ϕPGW were integrated at the landing site VK33 (Venken et al., 2006) on the third chromosome using ϕC31 integrase by Best Genes Inc. Similarly, ϕPGW carrying cDNA encoding the full-length Grip71 and Walium22 carrying shRNA targeting 5′ UTR or the coding sequence of Grip71 were integrated at attP2 (Groth et al., 2004) on the third chromosome.

### Immunological reagents and techniques

For generating an anti-Grip71 antibody, an entry vector (pENTR/D-TOPO) carrying the Grip71 full coding sequence from a cDNA (RE05579) was used. The cDNA has a mutation that leads to a premature stop codon after 493rd amino acid of Grip71. For expression of GST-tagged or MBP-tagged truncated Grip71 (Grip71*) in bacteria, the entry plasmid was recombined with expression vectors (pGEX4T1 and pMALc2) adapted to Gateway via LR Clonase (Invitrogen), respectively. *BL21(DE3) pLysS* containing GEX4T1-Grip71* were cultured overnight at 18°C in the presence of 1 mM IPTG. Bacteria were lysed by sonication in PBS + 0.5% Triton X-100 and spun down at 4,000 rpm for 5 min. The pellet contained most of GST-Grip71* which were largely insoluble in this condition. After a wash in the same buffer, the pellet was run on an SDS protein gel, and eluted from the gel by diffusion in 0.2M NaHCO_3_ + 0.02% SDS. A total of 2.5 mg of protein (four injections) was used to immunize a rabbit by Scottish National Blood Transfusion Service. The final anti-serum was then affinity-purified using MBP-Grip71* on nitrocellulose membranes as previously described (Smith and Fisher, 1984; Clohisey et al., 2014).

The following primary antibodies were used for immunofluorescence microscopy (IF) and immunoblots (IB) in the study: anti-GFP (rabbit polyclonal A11122; Thermo Fisher Scientific; 1:250 for IF and IB), anti-Subito (rat polyclonal against GST-Subito full-length; Loh et al., 2012; 1:250), anti-Grip71 (rabbit polyclonal affinity purified; this study; 1:2–1:10 for IF and 1:50 for IB), anti–γ-tubulin (mouse monoclonal GTU88; Sigma-Aldrich; 1:250 for IB), anti–α-tubulin (mouse monoclonal DM1A; Sigma-Aldrich; 1:250 for IF). Alexa 488-, Alexa 647-, Cy2- Cy3-, and Cy5-conjugated secondary antibodies were used (1:250 to 1:1,000; Jackson Laboratory or Molecular Probes) for IF, and IRDye 800CW conjugated goat anti-rabbit (1:20,000), IRDye 680LT conjugated goat anti-rabbit (1:15,000), and IRDye 800CW conjugated goat anti-mouse (1:20,000) secondary antibodies (LI-COR) were used for IB.

For immunoblotting, SDS-PAGE (sodium dodecyl sulfate PAGE) were performed and proteins on the gel were transferred onto a nitrocellulose membrane following a standard procedure (Sambrook et al., 1989). Total proteins on the membrane were stained with MemCode reversible staining kit (Thermo-Fisher) or Ponceau (Sigma-Aldrich). After being destained, the membrane was incubated with primary antibodies followed by fluorescent secondary antibodies (LI-COR). The signals were detected with an Odyssey imaging system for Fig. 2 B and Fig. S1 (B and E) and an Odyssey CLx imaging scanner (LI-COR) for Fig. 6 and Fig. S2. The brightness and contrast were adjusted uniformly across the entire area without removing or altering features.

For immunoblotting of ovaries, 10 ovaries or 100 oocytes were dissected from mature females in methanol. Methanol was replaced by 50 µl of water and 50 µl of boiling 2× sample buffer (50 mM Tris-HCl, pH6.8; 2% SDS; 10% glycerol; 0.1% Bromophenol Blue; and 715 mM 2-mercaptoethanol). Ovaries were homogenized in a microtube using a pestle (Eppendorf). The equivalent of 20 oocytes (20 µl; Fig. S1 E) or one ovary (10 µl; Fig. 2 D and Fig. S2) were loaded on a gel for SDS-PAGE followed by immunoblot. For S2 cells, 5 million cells were spun down at 500 *g* for 10 min, washed once with PBS at room temperature then resuspended in 50 µl of water and 50 µl of boiling 2× sample buffer. The equivalent of 500,000 cells were loaded on a gel for SDS-PAGE followed by immunoblot (Fig. S1 B).

### Cytology and image analysis

For immunostaining of mature oocytes, freshly eclosed females were kept with males for 3–5 d at 25°C with an excess of dried yeast. Ovaries from matured females were dissected in methanol and, after removing the chorion by sonication, oocytes were stained as previously described (Cullen and Ohkura, 2001). Under this experimental condition, most oocytes were arrested at stage 14.

Microtubule regrowth experiments in Fig. 4 (C–E) were performed as follows. 24 microtubes containing one to two mature females each were incubated on ice for 4 h to depolymerize microtubules. The absence of microtubules in oocytes after 4 h on ice was confirmed by immunostaining. Each tube was transferred one-by-one to water at 25°C, and after 2 min, ovaries were dissected in methanol and processed for immunostaining.

Immunostained oocytes were imaged with a confocal scan head, LSM510Exciter (Zeiss; Fig. 1 A; Fig. 2 A and C; Fig. 4 A; and Fig S1 C) or LSM800 with GaAsP photomultipliers (Zeiss; Fig. 3 and Fig. 4, B, D, and E), attached to an Axiovert 200M (Zeiss) using 63×/NA1.40 Plan-ApoChromat objective (oil) and 0.5-µm Z-step acquisition. The capturing resolution was set for 100 nm per pixel. Representative images are shown in figures after maximum intensity projection of multiple Z-planes including the entire spindle. The contrast and brightness of microscope images shown in the figures were unaltered. When the signal intensity is compared (Fig. 3, A and B), the images were taken using the same settings.

For *Drosophila* S2 cells (Schneider, 1972), approximately one million cells were aliquoted into each well of a 6-well Petri dish with 1 ml of Serum Free Medium (Schneider). 15 µg of dsRNA is added to each well. After 1 h, 2 ml of the medium containing 10% FCS was added to the cells. Alternatively, between 1 and 3 million cells were transfected with 0.4 µg of inducible pMTGW-Subito WT or pMTGW-Subito-NLS* using Effectene Transfection Reagent (Qiagen) following the manufacturer’s instructions, in the presence of 0.5 µM of CuSO_4_ to activate the metallothionein promoter. Three days later the cells were transferred to concanavalin A coated coverslips (18 × 18 mm; VWR), fixed with a cold solution containing 90% methanol, 2.88% paraformaldehyde, and 5mM NaHCO_3_ (pH9), and stained as previously described (Dzhindzhev et al., 2005). A single Z section of S2 cells was imaged with a charge-coupled device camera (Orca; Hamamatsu) attached to an upright microscope (Axioplan2) controlled by OpenLab (Improvision) using a 100×/NA1.4 Plan-ApoChromat objective.

For Fig. 3, the total tubulin intensity was measured on spindles imaged with the same setting (A). If some signals were saturated, a second image was taken with a lower laser intensity (B). To compensate for the lower laser power, a laser coefficient was calculated by the ratio (A/B) of the total signal intensity of three spindles without saturated signals taken by these two settings. Tubulin signal intensity was measured from maximum intensity projection of a z-stack. A region S mainly containing the spindle was drawn, and the total intensity (IntDenS) and the area size (AreaS) was measured. A second region L containing the spindle and the surrounding background was drawn, and the total tubulin intensity (IntDenL) and the area size (AreaL) were measured. The following formula was then applied to calculate the total tubulin intensity for each spindle: IntDenS-AreaS × (IntDenL-IntDenS) / (AreaL-AreaS). For the overexposed spindles, the tubulin intensity was measured from an image captured using setting B and the following formula was applied: LaserCoefficient × [IntDenS-AreaS × (IntDenL-IntDenS) / (AreaL-areaS)]. Two-tailed Wilcoxon rank-sum test was used for testing statistical significance.

For Fig. 4 E and Fig. S1 D, the tubulin and Grip71 signal intensities were measured from the maximum intensity projection of a Z-stack as follows. A small region of interest (region C) was drawn on the ectopic spindle (Fig. 4 E) or near the chromosomes on the spindle (Fig. S1 D), and the total signal intensities of tubulin and Grip71 were measured. A region of interest with the identical shape and size (region D) was drawn in a background region and the total signal intensities were measured. The specific signal intensity was calculated by subtracting the total intensity of region D from the total intensity of region C. For Fig. S1D, the specific signal intensity of Grip71 was then normalized by dividing it by the specific tubulin signal intensity of the same spindle. Two-tailed Wilcoxon rank-sum test was used for testing statistical significance.

For Fig. 1 B, Fig. 2 E, and Fig. 3 C, accumulation of Grip71 and Subito in the spindle equator and poles was judged in comparison with the intensity distribution of tubulin signals by visual inspection of each z-plane. To check the validity of classification, a series of raw images on which Fig. 1 B is based were independently examined by another researcher in a blind manner, which gave a similar result. Multiple independent batches of immunostaining were performed for each genotype, and the mean and SEM were calculated for the percentage of spindles which showed the accumulation in each replicate. Two-tailed unpaired *t* test was used for testing statistical significance.

For Fig. 2 A, the fluorescence signal intensity for each channel was measured along the line drawn on the equator of the WT spindle shown on the left, after the maximum intensity projection onto the xy plane was performed. To assist the visual comparison of the signal distribution between the channels in the same graph, the intensity value of the Subito signals in an arbitrary unit was multiplied by 1.2.

For Fig. 1 C and Fig. 2 F, bipolar spindles were chosen for each condition and analyzed in ImageJ (National Institutes of Health). For each spindle, maximum intensity z projection was performed and the image was rotated in a clockwise manner to put the spindle horizontally. The image was cropped to a rectangle box of 30 × 10 µm with the middle of the box (15 µm) being aligned with the center of the chromosome mass. After each channel was separated, nonspecific bright speckles outside of the spindle area were manually removed from the Grip71 channel by drawing circles and clearing the signals inside. Each channel was resliced from xy to xz without interpolation. Maximum intensity z projection of the newly obtained stack was performed and a plot profile was generated for each channel. For each spindle, the average background intensity was obtained by averaging signal intensities of the first and last 5-µm region. To calculate a normalized signal intensity, the average background intensity was first subtracted from the signal intensity at each point along the spindle and then this specific signal intensity was divided by the average background intensity. The graphs in Fig. 1 C and Fig. 2 F represent the averages of 21 and 14 spindles for each condition from triplicated and duplicated experiments, respectively. The error bar represents the SEM of the normalized intensity at each point along the spindle axis. Unpaired *t* test was used for testing statistical significance of differences in the signal intensity in the equatorial region of the spindle (the mean intensity in the region between −1 and +1 µm).

For Fig. 4, B and D and Fig. S4 E, the entire oocytes were observed for the presence or absence of dense microtubule arrays not associated with the meiotic chromosomes (“ectopic spindles”). Two independent batches of immunostaining were performed for each genotype in Fig. 4 D and three independent batches in Fig. S4 E. The mean and SEM were calculated for the percentage of oocytes which have visible ectopic spindles in each replicate. Two-tailed unpaired *t* test was used for testing statistical significance. As only one batch of immunostaining was performed for GFP-SubitoΔN + *white* shRNA in B, statistical significance was tested by Fisher's exact test.

For Fig. S4 C, two independent batches of immunostaining were performed, and the mean and SEM were calculated for the percentages of cells displaying GFP in the nucleus. Unpaired *t* test was used for testing statistical significance.

### Immunoprecipitation and microtubule nucleation assay

Immunoprecipitation and microtubule nucleation assay shown in Fig. 5, Fig. 6, and Fig. S3 were performed as follows. Flies expressing GFP, GFP-Grip71, GFP-Subito WT + *white* shRNA, GFP-Subito WT + *Grip71* shRNA, GFP-SubitoΔN + *white* shRNA, or GFP-SubitoΔN + *Grip71* shRNA were matured with dried yeast for 3–5 d at 25°C. 20 pairs of ovaries from each genotype were dissected in PBS + 2 mM EGTA and frozen with a minimum carry-over of the buffer on the side of a microtube prechilled on dry ice. Microtubes were snap frozen in liquid nitrogen and stored at −80°C.

For immunoprecipitation, 20 pairs of frozen ovaries were resuspended in 400 µl of lysis buffer (20 mM Tris-HCl pH7.5, 50 mM NaCl, 5 mM EGTA, 1 mM DTT supplemented with 1 mM PMSF, and inhibitor of protease [Roche, 1 tablet for 10 ml]) + 1 µM okadaic acid, 10 mM p-nitrophenyl phosphate and 0.5% Triton X-100 and homogenized on ice with a Dounce tissue grinder (1 ml). Lysates were incubated on ice for 30 min and transferred in a microtube before being centrifuged for 30 min, at 13,000 rpm at 4°C. 10 µl was kept as Input and mixed with 10 µl of boiling 2× sample buffer, and the rest was used for the following immunoprecipitation. 11 µl of anti-GFP magnetic beads (Chromotek) were washed three times with cold lysis buffer + 0.1% Triton X-100 before being mixed with lysate supernatant for 1 h at 4°C on a rotating wheel. The beads were washed once with cold lysis buffer containing 0.1% Triton X-100, 1 µM okadaic acid, and 10 mM p-nitrophenyl phosphate. The beads were additionally washed twice with cold lysis buffer + 0.1% Triton X-100 and twice with cold BRB80 (80 mM PIPES, 1 mM MgCl_2_, and 1 mM EGTA, pH 6.8). The beads were resuspended in 10 µl of cold BRB80 and kept on ice for the following microtubule nucleation assay.

For nucleation assay, pig unlabeled pure tubulin (Cytoskeleton T240-B) and pig rhodamine-labeled pure tubulin (Cytoskeleton TL590M-A) were resuspended in BRB80 + 1 mM GTP to a final concentration of 50 µM and 100 µM, respectively. Unlabeled and labeled tubulin were mixed (4.5:1 in molar concentration) to a final concentration of 55 µM then diluted in BRB80 + 1 mM GTP to a final concentration of 36.6 µM. 9 µl of this mix was added to 1 µl of the beads for a final concentration of 33 µM of tubulin. The beads and tubulin were incubated for 3 min at 37°C to allow polymerization. To stop the reaction, 10 µl of BRB80 at 37°C containing 1 mM GTP and 2% glutaraldehyde (G6403; Sigma-Aldrich) was added to 10 µl of the reaction and gently mixed using a pipet tip, the end of which has been cut off. 10 µl of the reaction was mixed with 10 µl of BRB80 + 30% glycerol (15% glycerol final) to increase the viscosity and reduce movement of the beads. 4 µl of the reaction was mounted between two coverslips (24 × 50 mm and 18 × 18 mm, VWR).

Images were taken using a PlanApochromat lens (63×/1.4 NA; Zeiss) attached to an Axiovert 200M microscope (Zeiss) with a spinning disc confocal scan head (CSU-X1; Yokogawa) operated by Volocity (PerkinElmer). 20–40 random fields were imaged with 488-nm laser (20%) to detect the GFP beads and 561-nm laser (20%) to detect the rhodamine labeled microtubules (250-ms exposure, 0.8 µm z-interval). Microtubules associated with each bead cluster were counted from the maximum intensity projection of z-stack. The numbers of free microtubules were counted in the single z-plane where the GFP intensity was the highest (Fig. S3). Two-tailed unpaired Wilcoxon rank-sum test was used for testing statistical significance. The images were taken using the identical setting and are shown in Fig. 5 B after applying the identical contrast and brightness enhancement. After the experiment, ∼10 µl of the remaining beads were mixed with 10 µl of boiling 2× sample buffer. 10 µl of Input and beads (IP) were loaded on a gel for SDS-PAGE followed by immunoblot (Fig. 6).

Quantification of Fig. 6 was performed as follows. Each membrane was imaged with an Odyssey CLx (LI-COR) which normalized the laser intensity of both the 700-nm and 800-nm channels allowing cross-comparison between membranes. A box was drawn around each band of interest. The background was automatically subtracted from the total signal intensity using the border tool that calculates the median background of each band using a small box of 3 pixels on the top and 3 pixels on the bottom of the defined selection. For normalization, this specific total signal of each band in the IP lane was divided by the specific signal of the γ-tubulin band (obtained in the same manner) in the corresponding input lane. The normalized signals in three independent experiments were averaged and plotted on the graph. Error bars represent the SEM. Unpaired *t* test was used for testing statistical significance. The same quantification method was used in Fig. S2, except that the signal of each band of interest was normalized against the intensity of the yolk protein band cross-reacted by anti-Grip71 antibody in the corresponding input lane. The normalized signals in three independent experiments were averaged and plotted on the graph. Error bars represent the SEM. Unpaired *t* test was used for testing statistical significance.

Immunoprecipitation shown in Fig. 2 B was performed as follows. Ovaries were dissected from matured females for each condition in PBS + 0.5% Triton X-100. 25 pairs of ovaries were resuspended in 500 µl of lysis buffer. Ovaries were homogenized on ice using a Dounce tissue grinder (1 ml). Lysates were incubated on ice for 30 min and transferred in a microtube before being centrifuged at 13,000 rpm for 30 min at 4°C. 50 µl of the supernatant was kept as Input and mixed with 50 µl of boiling 2× sample buffer, and the rest is kept for the following immunoprecipitation. 30 µl of anti-GFP magnetic beads were washed three times with cold lysis buffer + 0.1% Triton X-100 before being mixed with the lysate supernatant for 2 h at 4°C on a rotating wheel. The beads were isolated with a magnetic rack and washed three times with cold lysis buffer + 0.1% Triton X-100. The beads were resuspended in 50 µl of cold lysis buffer + 0.1% Triton X-100 and mixed with 50 µl of 2× boiling sample buffer. 10 µl of the Input and beads (IP) were loaded on a gel for SDS-PAGE followed by immunoblot.

### Online supplemental material

Fig. S1 shows the specificity of the anti-Grip71 antibody Fig. S2 shows Grip71 and Subito protein levels in the absence of each. Fig. S3 shows that SubitoΔN beads induce microtubule nucleation rather than simply capture microtubules. Fig. S4 shows that the NLS in the N-terminal region of Subito is not required for suppression of the Subito activity to induce ectopic microtubule arrays.

## Supplementary Material

Supplemental Materials (PDF)
