# Supplementary material for: A novel microtubule nucleation pathway for meiotic spindle assembly in oocytes
Source: J Cell Biol. 2018 Oct 1;217(10):3431–45. doi: 10.1083/jcb.201803172 (PMC6168254; doi:10.1083/jcb.201803172)
Supplement: Supplemental Materials (PDF) [file JCB_201803172_sm.pdf]

Supplemental material

Romé and Ohkura, https://doi.org/10.1083/jcb.201803172

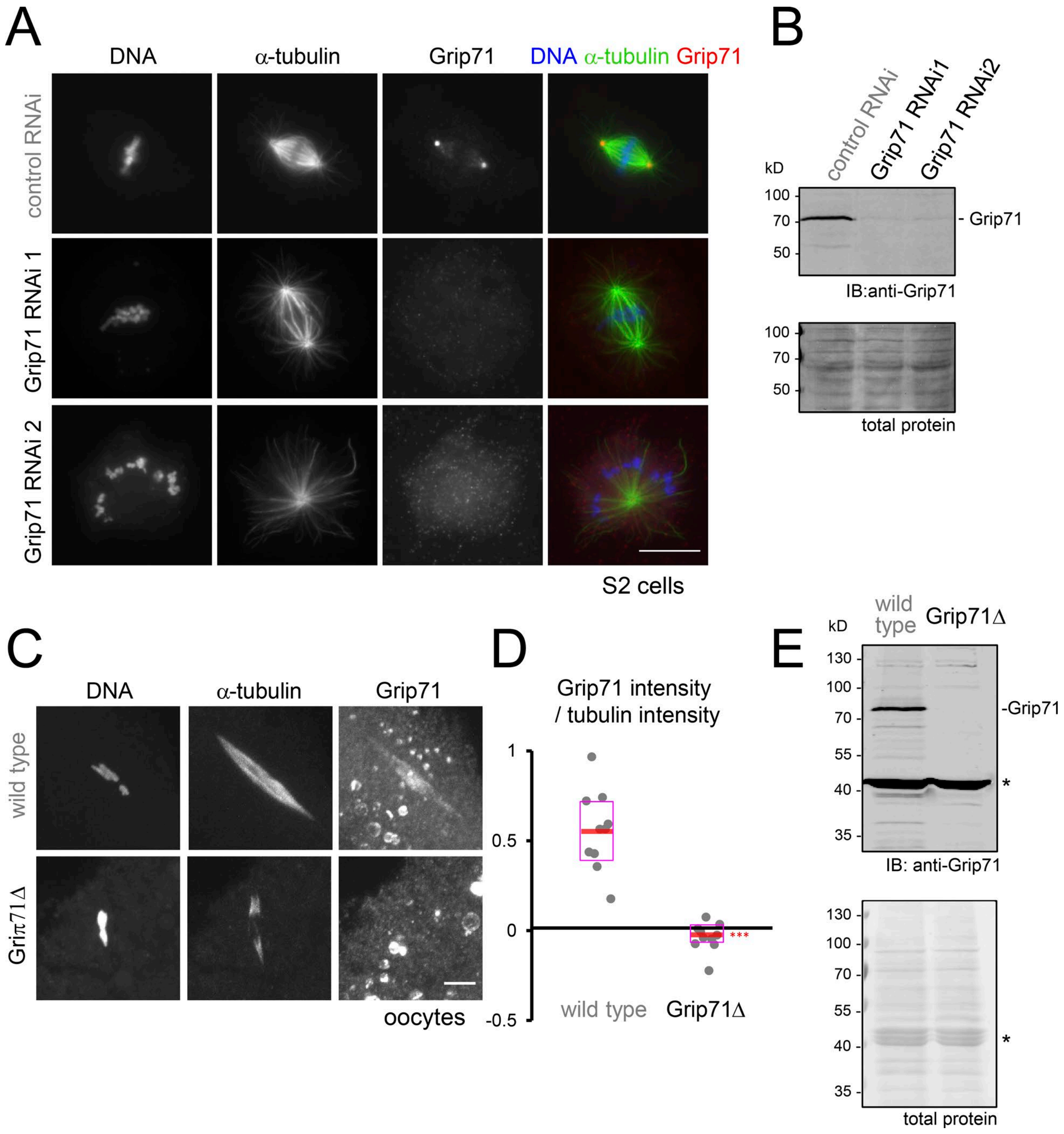

Figure S1. **Specificity of the anti-Grip71 antibody. (A)** Immunostaining of S2 cells incubated with control dsRNA (against β-lactamase gene) and two nonoverlapping dsRNA against *Grip71*. The rabbit antibody against Grip71 stained spindle poles in control RNAi, but the signal greatly reduced in Grip71 RNAi cells. Bar, 10 µm. **(B)** Immunoblot of S2 cells after RNAi using the anti-Grip71 antibody. The anti-Grip71 antibody gave a band of the expected size, the intensity of which was greatly reduced following Grip71 RNAi. **(C)** Immunostaining of a meiotic spindle in mature oocytes from WT and a Grip71 deletion mutant using the anti-Grip71 antibody. Bar, 5 µm. **(D)** Grip71 signal intensity relative to α-tubulin signal intensity near the chromosomes on the spindle in mature WT and *Grip71Δ* oocytes (10 spindles each). The signal intensity on the spindle in each oocyte was normalized using the background (cytoplasmic) intensity. The median is indicated by the central line, and the second and third quartiles are indicated by the box. The graph shows the data from one of two repeated experiments, both of which showed similar decreases in *Grip71Δ* oocytes. *** indicates a significant difference from WT ($P < 0.001$; Wilcoxon rank sum test). **(E)** Immunoblot of ovaries from WT and Grip71 deletion mutant probed by the anti-Grip71 antibody. The 75-kD band disappeared in the deletion mutant and roughly coincides with the predicted molecular weight of Grip71, indicating that this band represents Grip71. On the other hand, the 42-kD band (*) that coincides with yolk proteins was unchanged, indicating that this band is nonspecific.

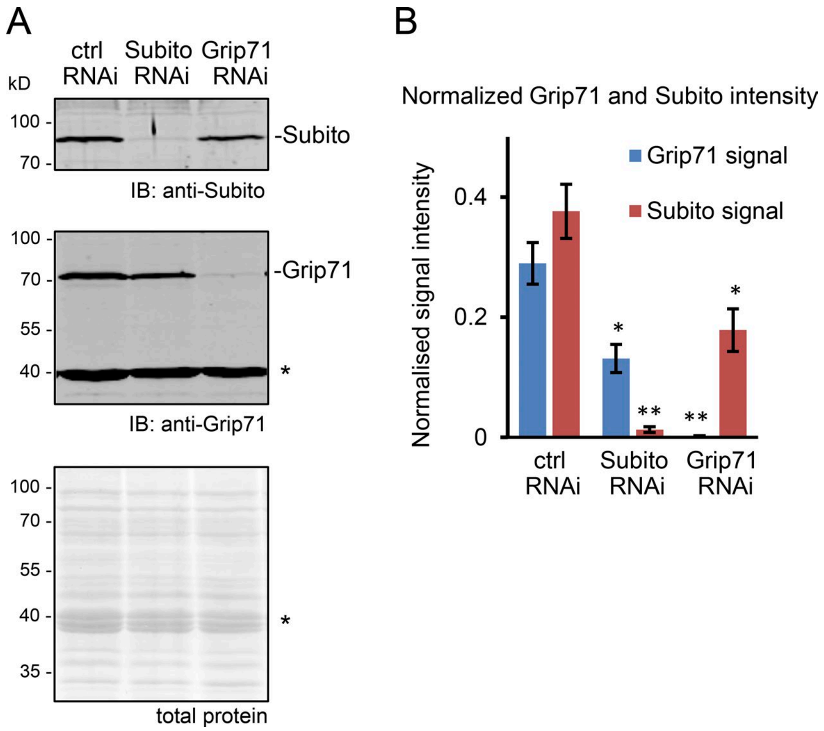

Figure S2. **Grip71 and Subito protein levels in the absence of each. (A)** Immunoblots of ovaries from control (*white*), *subito*, and *Grip71* RNAi flies, with total protein staining. * indicates the nonspecific band recognized by the anti-Grip71 antibody, which corresponds to the position of yolk proteins. **(B)** The means and SEM of normalized Grip71 and Subito signal intensities from triplicate immunoblots. The intensity of the nonspecific band was used for normalization. * and ** indicate significant differences from control RNAi ($P < 0.05$ and $P < 0.01$, respectively; *t* test).

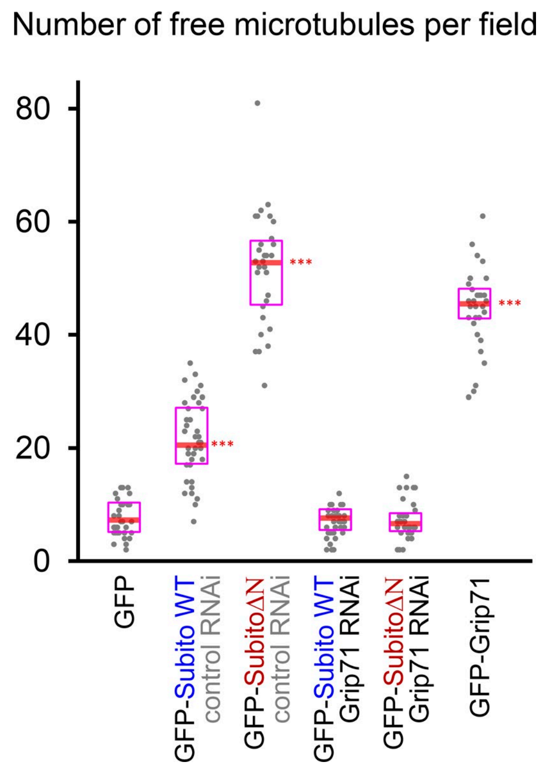

Figure S3. **SubitoΔN beads induce microtubule nucleation rather than simply capture microtubules.** The number of free microtubules observed per field (51 × 102 µm) in the in vitro microtubule nucleation assay shown in Fig. 5 using immunoprecipitated beads and pure α,β-tubulin dimer (35, 40, 30, 39, 35, and 34 fields). The median is indicated by the central line, and the second and third quartiles are indicated by the box. *** indicates a significant difference from WT ($P < 0.001$; Wilcoxon rank sum test). The graph shows the data from one of two repeated experiments, both of which provided similar results. A solution including GFP-SubitoΔN beads had much more free microtubules than one including control GFP beads, confirming that the GFP-SubitoΔN beads induced nucleation of microtubules (some of which were detached from the beads), rather than simply captured microtubules spontaneously nucleated in solution.

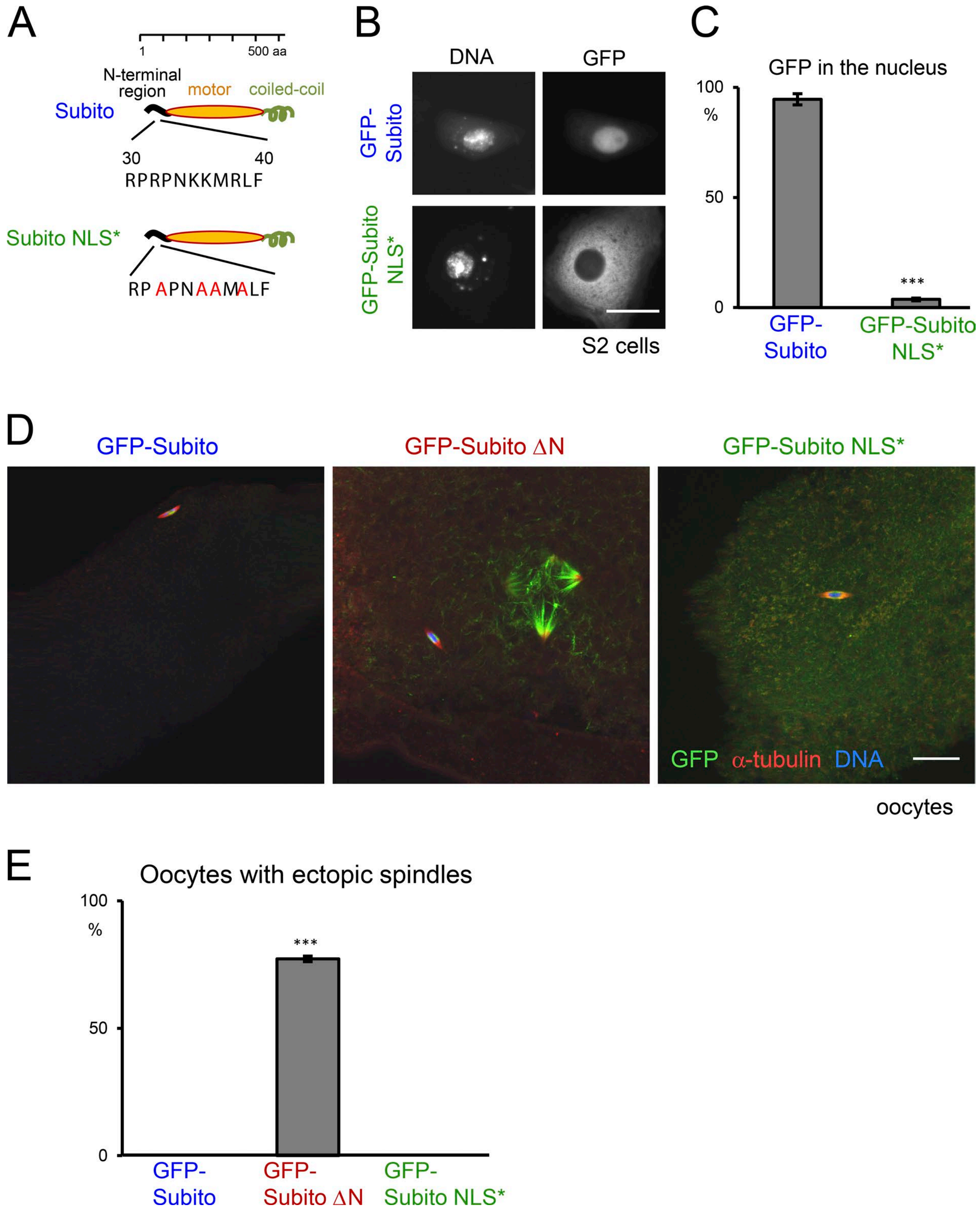

Figure S4. **The NLS in the N-terminal region of Subito is not required for suppression of the Subito activity to induce ectopic microtubule arrays. (A)** A diagram representing the NLS identified with cNLS mapper in the N-terminus of Subito. R32, K35, K36, and R38 have been mutated into alanines to impair the function of the NLS. **(B)** Immunostaining of S2 cells transiently expressing GFP-Subito or GFP-Subito NLS*. GFP (Subito) and DNA were stained. GFP-Subito is concentrated in the nucleus, while Subito NLS* is diffused in the cytoplasm. Bar, 10 µm. **(C)** Frequencies of cells showing a nuclear localization of Subito and Subito NLS*. The graph shows the means and SEM from duplicated experiments (totals of 277 and 324 cells, respectively). **(D)** Immunostaining of mature oocytes expressing GFP-Subito, GFP-SubitoΔN, or GFP-Subito NLS*. GFP (Subito), α-tubulin, and DNA were stained. Bar, 15 µm. Only GFP-SubitoΔN induced ectopic spindle formation in the cytoplasm. **(E)** Frequencies of oocytes with ectopic spindles. The graph shows the means and SEM from triplicated experiments (totals of 566, 472, and 540 oocytes, respectively). *** indicates a significant difference from GFP-Subito ($P < 0.001$; *t* test).
